# Republication of English Works

**Published:** 1854-03

**Authors:** 


### REPUBLICATION OF ENGLISH WORKS.

Our learned correspondent, in his remarks on reprints of English works by American publishers and their indiscriminate praise by our medical journals, is hardly just, we think, to those who have control of the medical press. No doubt the laudation is quite too general, and that the system is injurious to our native literature; but we are sure that our able correspondent has not hit upon the true explanation of it. The real cause is not selfishness, but indolence; it is easier to praise a book in general terms than to criticise it intelligently, and this is the chief reason why the praise of foreign works is so indiscriminate. We take their merits, for the most part, upon trust, without thorough, much less critical, examination. We are free to confess our short-comings in this particular, and suppose most of our brethren would have to own, that, in the press of other urgent duties, they have sometimes been tempted to commend hastily. But there is this to be said in extenuation of the practice, which certainly cannot be justified, that the wheat which comes to us across the Atlantic has generally been thoroughly winnowed at home, and but little of the chaff is likely to reach our shores. It is a rare mistake for one of our shrewd publishers to fall upon a worthless English book. They have had the benefit of all the light of the English periodical press, and seldom venture upon a republication until the book has run the gauntlet at home. Still, we agree with our correspondent, that we ought not to be contented with this state of things. It is time we had declared our independence of the mother country in this particular also, and as a profession had set up for ourselves. Let us encourage our own writers; let us foster our own indigenous literature.

